# Do AI-based contouring algorithms influence physicians in the online adaptive radiotherapy of patients with bladder cancer?

**DOI:** 10.1016/j.tipsro.2026.100401

**Published:** 2026-04-13

**Authors:** Nika Guberina, Aymane Khouya, Christian Hoffmann, Gerrit Fischedick, Yasemin Alberti, Julian Hlouschek, Fabian Freisleben, Andreas Herz, Mike That Troung Ton, Lars Oliver Kiwitt, Ezgi Ceren Sahin, Alina Santiago Garcia, Thomas Gauler, Christoph Pöttgen, Maja Guberina, Martin Stuschke

**Affiliations:** a Department of Radiotherapy, West German Cancer Center, University Hospital Essen, University of Duisburg-Essen, Germany; b German Cancer Consortium (DKTK), Partner Site University Hospital Essen, Germany

**Keywords:** Artificial intelligence, Human machine interaction

## Abstract

**Purpose:**

In this prospective cross-over study, the precision of manual correction of the clinical target volume (CTV) during online-adaptive radiotherapy (ART) of patients with bladder cancer was investigated in dependence on the input of applied computational algorithms.

**Methods and materials:**

Online-adaptive-radiation therapy (ART) fractions were analysed from a prospective registry as a component of a trimodality treatment for bladder cancer. The CBCT-guided adaptive radiation therapy (CBCTgART) workflow uses a CBCT acquired at the beginning of a treatment session (CBCT1) to update the target volume contours of the planning CT (pCT) on the current anatomy of the day. The study comprises radiotherapy fractions with target (CTV) segments showing clinically relevant deviations (>5 mm) when mapped between pre-treatment (CBCT1) and post-adaptation (CBCT2) images. CTV_algo1-2_ were obtained from two deformable-image-registration algorithms (algo_1,_ algo_2_) based on AI-generated normal-tissue contours and a rigid copy of the CTV_original_ termed CTV_algo3_ (algo_3_). Verification cone-beam-computed-tomography (CBCT2) acquired during ART showed CTV_algo1-3_ on new anatomical scenarios. The task of 13 physicians was to adjust CTV_algo1-3_ on CBCT2 to match the CTV_original_ on CBCT1.

**Results:**

In 151/169 pairwise comparisons, mean-distance-of-segments-to-ground-truth (MDA_GT_) after adjustment by a physician was larger for the poorer algorithms than for the better ones. The binomial proportion was 0.893, significantly different from 0.5 as null proportion (p < 0.0001, exact-binomial-test), and not dependent on the physician or on the respective patient. In a quantitative analysis by a mixed-model, the MDA_GT_ of the manually adjusted segments between 16% and 50% of the errors were on average corrected by the physicians, with interindividual differences (p < 0.0001,F-test).

**Conclusions:**

The present study results reveal that the quality of AI-generated contours is of crucial importance for a proper online onboard ART, as errors may bias clinical workflow and contouring. The accuracy of supervised structures strongly depends on the accuracy of the AI generated contours before supervision.

## Highlights

• The accuracy of supervised structures depends on the quality of AI-generated contours.
• Errors by contour generating algorithms may bias final treatment schedules.
• The worse algorithm delivered the worse result after review and adjustment.

## Introduction

Online adaptive radiotherapy (ART) is a newly developing technology, aimed at enhancing the precision of radiotherapy, with the potential to reduce planning target volume (PTV) margins around the clinical target volume (CTV) and thereby to spare normal tissue. If the process of online adaptation is fast, online adaption can lead to reduced PTV margins to maintain a clinically good dose coverage of the CTV, determined on the post-adaptation anatomy captured with a verification CBCT (CBCT2) [1]. Bladder cancer is one of the tumor entities, where PTV margins >10 mm around the CTV are used for standard image guided radiotherapy (IGRT) in order to cover the mobile CTV with the prescribed dose [2]. The fact that a reduction in the PTV margin can reduce the bladder and gastrointestinal side effects of pelvic radiotherapy has been shown in randomised studies for prostate cancer [3], [4]. ART is available on linear accelerators, equipped with cone beam computed tomography (CBCT) or magnetic resonance imaging (MRI). Both devices, the MR-linac as well as the CBCT-based radiotherapy system have received a 510(k) clearance as class II devices from the US Food & Drug Administration. They are therefore determined as compatible with their predecessor devices marketed prior 1976. The accuracy of the deformed organs and structures of the day and the quality of the adapted plan have to be supervised by authorized personal and these decisions are not performed autonomously by the systems. The AAPM Task group No. 132 on the use of image registration and fusion algorithms in radiotherapy and a current review from NRG oncology states that a final review and validation of image registration for clinical use should be performed by the treating radiation oncologist and residual errors should be corrected or accounted for by treatment margins [5], [6]. Current FDA-approved or CE-marked treatment systems for online adaptive radiotherapy are intended as assistant tools rather than as autonomous unsupervised systems [7]. These systems require AI-generated structures to be reviewed and approved by qualified clinicians and new treatment plans to be approved by both a qualified physician and a medical physicist [8], [9], [10]. Thus, for these assistive devices a close interaction between physicians and the device is required.

Here in the present study, a CBCT-guided adaptive radiation therapy (CBCTgART) workflow is used. Target volume contours are updated using deformable image registration of the planning CT (pCT) on the CBCT acquired initially at the beginning of a treatment session (CBCT1). This process is performed in the pelvis with the help of AI-based segmentation of surrounding organs or influencer structures [8]. After supervision of the AI-generated structures by a radiation oncologist, these influencer structures are used for a structure-guided deformable registration to propagate the target volume from the pCT on the initial CBCT of the day [8]. This target volume is then reviewed by the radiation oncologist and used for online optimizing of the new adaptive plan to the anatomy of the day [5], [11], [12]. There is currently limited research examining, how physicians respond to AI-generated advice or suggestions of ART. Previous research indicates that AI users may tend to rely on such advice and find it difficult to ignore it, even if it is inaccurate. This phenomenon has been observed not only in medical contexts among physicians [13], [14], but also in various other important decision-making situations [15].

The underlying hypothesis of the above ART workflow is that the human reader, here the radiation oncologist, can review the machine-generated structures in an unbiased way and precisely correct the structures by comparing the new imaging study with the original reference imaging study side by side. While there are examples from the field of medicine where physicians and AI-algorithms interact effectively [16], [17], there are also examples from health contexts, where errors of AI-based devices biased the supervising physicians [14], [18], [19]. Thus, careful consideration must be given to potential biases introduced by these technologies. The primary aim of this study is to examine the risk of bias, that an error caused by an algorithm is accepted or only partially corrected.

## Material and methods

### Study design

Online adaptive radiation therapy (ART) fractions were obtained from a prospective registry as a component of a trimodality treatment for bladder cancer. As study design we chose a prospective cross-over study. We examined the precision of altogether 13 physician’s manual correction of the clinical target volume (CTV) during online-adaptive radiotherapy (ART) of patients with bladder cancer in dependence on the input of applied computational algorithms.

### Patient selection

The prospective registry comprised 9 patients who underwent ART after transurethral resection of bladder tumor (TURBT) and after intravesical instillation of chemotherapy. The multimodal, primarily organ-preserving therapy was offered to patients with locally confined, muscle-invasive urothelial carcinoma (cT2-4 cN0/NX M0) not suitable for radical cystectomy or to those patients who were seeking an alternative to radical cystectomy according to the decision of an interdisciplinary tumor board. Contouring was performed following institutional guidelines as described in [20].

### Ethical considerations

All treated patients gave their consent to the treatment taking part in the prospective, institutional clinical registry trial (18-8364-BO). All participating physicians gave their informed consent to participate in this prospective study approved by the Ethics committee of University Hospital Essen of University of Duisburg-Essen (23-11595-BO). The study was conducted in accordance with the principles of the Declaration of Helsinki.

### Treatment procedures

The study analyzed 213 radiotherapy fractions to identify localized bladder target (CTV) segments showing clinically relevant deviations (>5 mm) when mapped between pre-treatment (CBCT1) and post-adaptation (CBCT2) images using different deformable image registration methods. Small, anatomically anchored segments were selected across multiple bladder regions and used as ground truth references by aligning them rigidly between scans. Three propagation approaches were tested: (I) a contour-based deformable algorithm, (II) a hybrid intensity- and structure-based algorithm, and (III) a rigid copy method. For each case, physicians, blinded to the algorithm and patient details, reviewed and manually adjusted the propagated target volumes on CBCT2 to match the original anatomy seen on CBCT1 within a simulated clinical workflow. These adjustments were performed across multiple sessions with randomized presentation of cases and algorithms. The accuracy of each method after physician correction was then evaluated by measuring the mean distance between the adjusted segments and the predefined ground truth, allowing comparison of algorithm performance based on how closely corrected contours matched the true anatomical reference*.*

### Identification of CTV segments

For analysis, various anatomical scenarios were identified, obtained from 137 different dose fractions of focal bladder radiotherapy and 76 fractions of whole bladder radiotherapy with or without pelvic lymph node regions at the ETHOS therapy system. All these 213 dose fractions were screened by the steering group of this study [MS and AK], not involved with the adaption task. The aim of this screening was to identify test segments on the pre-fraction CTV during online adaptive radiotherapy that exhibit clinically relevant deviations when deformed from the pre-treatment CBCT1 to the post-adaptation verification cone-beam CT (CBCT2) using different deformable image registration (DIR) algorithms. Deviations were considered clinically relevant when the shortest distance between the deformed test segments exceeded 5 mm. The selected test segments were intended to represent the full range of clinically relevant deviations produced by the evaluated DIR algorithms across the entire dataset. The selected segments had to be local and therefore their surface area had to be smaller than 25 cm^2^, but larger than one cm^2^. CTV-segments should cover deformations in all octants defined from the bladder center as inferior-anterior, inferior-posterior, inferior-right-lateral, inferior-left-lateral, and the respective superior octants. The identified segments covered CTV deformations in 6 of the 8 octants in relation to the center of the bladder. Octants not covered by the segments were the superior-right-lateral and superior-left-lateral octants. In addition, the CTV segments had to be located near anatomical landmarks, such as the bony borders of the symphysis, prostatic calcifications, or anatomical boundaries in tissues surrounding the bladder or the bladder wall adjacent to the prostate, to enable a local rigid registration between CBCT1 and CBCT2 for ground truth determination. The CTV1 near the landmarks was copied from CBCT1 to CBCT2 as local ground truth for the respective nearby segment of CTV2.

### Study design and computational algorithms

**Fig. 1:**
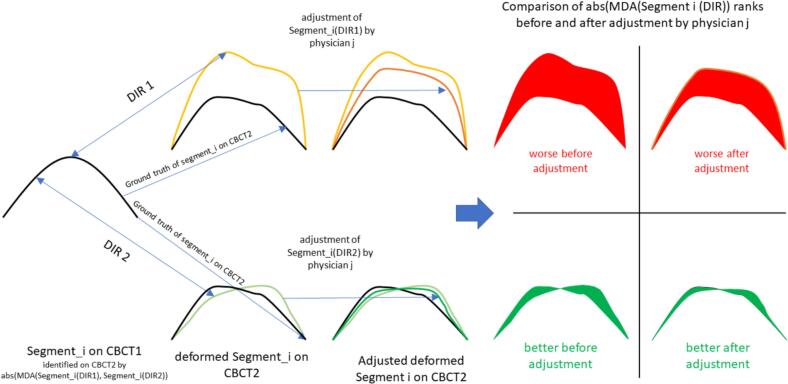
Flow chart of trial activities. The deformed CTV on CBCT2 propagated by two deformable image registration algorithms (DIR) and in addition a rigid algorithm were presented to 13 physicians in random sequence in three sessions. The physicians had to adapt the CTV and the accuracy of this adaptation was analysed in segments, in which the surface of the CTV propagated by both algorithms differed by more than 5 mm.

From the selected dose fractions, a pre-adaptation CBCT (CBCT1) of a treatment session and a second verification CBCT after online plan adaptation (CBCT2) were obtained. The structure set for CBCT1 contained the reference clinical target volume (CTV_1_) for that session, bladder, and rectum reference structures initially generated by the online AI-tool of the Ethos® system (Varian, Palo Alto) as well as supervised and corrected by a radiation oncologist. Bladder and rectum structures for this study on CBCT2 were generated by using the AI-based segmentation tool in MIM ProtégéAI® (MIM Software Inc., Cleveland, OH, USA, version 1.1.3) of MIM Maestro®software (version 7.3.2, MIM Software Inc., Cleveland, OH, USA). Three approaches were employed for propagation of the reference CTV_1_ from CBCT1 to CBCT2 (CTV2_algo1-3_). Algorithm 1 (algo_1_) was a contour-based DIR algorithm with bladder and rectum as a constraint term from the VoxAlign Deformation Engine® within MIM Maestro®, algorithm 2 (algo_2_) used a hybrid deformable registration considering both, intensity values as well as bladder and rectum, and method 3 (algo_3_) was a 1:1 copy algorithm relied on the clinically generated rigid registration (Fig. 1). The test segments were identified using the MIM Maestro software by using Boolean operations. First, CTV2_algo1_ was expanded outward by 1 mm and then subtracted from the original CTV2_algo1_, creating a surface rim. This rim was intersected with CTV2_algo2_, which had been contracted inward by 5 mm. Corresponding surface segments were constructed on CTV2_algo2_. These segments were then back-transformed to CTV1 by the respective inverse transformations of algorithm 1 or algorithm 2. Once the test segments were defined on CTV1 in CBCT1, they were propagated to CTV2 using algorithms 1–3 prior to physician correction, as well as using CTV-based deformation after physician adaptation. For the latter, the adapted CTV2 and CTV1 served as the sole corresponding structures for deformation.

### Task of physicians

The task of the participating physicians was to adapt CTV2_algo1-3_ on CBCT2 so that it corresponded anatomically to the reference CTV1 on CBCT1 alike to the online adaptive workflow of the ETHOS therapy system in the clinical routine. CBCT2 with the deformed CTV2_algo1-3_ as well as CBCT1 with the reference CTV_1_ was presented side-by-side to each physician on the MIM Maestro program graphical user interface with tumor-specific information. The physician was blinded to both patient identifiers and the specific algorithm algo1-3 employed in generating the CTV2_algo1-3_ on CBCT2. Presentation of each CTV2_algo1-3_ generated by one of the three algorithms was planned for each physician during three different reviewing sessions within 3 months. In each session, 10 CTV2 _algo1-3_ structures on CBCT2 studies from 10 different dose fractions from ten different treatment series were presented to the physician, along with bladder and rectum contours by the same medical physics expert. Three CTV2 structures contained 2 different test segments. Within each reviewing session, physicians had a maximum of 100 min time to review and adjust the 10 anatomical scenarios. In a prospective cross over trial, CTV2_algo1-3_ from different treatment sessions were randomly assigned to physicians across three sessions, so that each physician supervised CTV-structures from all selected dose fractions for presentation by a randomly permutated algorithm in a random sequence per session (Proc plan, SAS). The frequency how often a deformation by a considered algorithm was preceeded by another algorithm was balanced. The physician was blinded to both, patient identifiers and the specific algorithm algo1-3, employed in generating the CTV2_algo1-3_ on CBCT2. A simultaneous truth and performance level estimation (STAPLE) consensus volume was constructed from the adjusted CTV2 volumes for each algorithm separately using the MIM software [21].

### Primary endpoints and null hypothesis

The primary endpoint of this study was the examination of the mean distance to agreement (MDA) between a test segment from the CTV2_algo1-3_ contour after manual correction and the local ground truth test segment surface (MDA_GT_). The absolute value of MDA_GT_ between CTV2_algo1-3_ segments and the respective ground truth segment were calculated using the MIM Maestro software. In addition, MDA_GT_ values were assigned a positive or negative sign based on whether the test segment from CTV2_algo1-3_ exhibited inward or outward deformation from the ground truth with respect to the centre of mass of the CTV2. This sign indicates the directionality of the resulting MDA_GT_ (dMDA_GT_). The null hypothesis for this study (H0) was that after correction by the supervising physician the MDA_GT_ between a corrected segment of CTV2_algo1_ or CTV2_algo2_ and the ground truth segment are equally small, thus, independent from the algorithm. For each given segment, the respective algorithm that produced the smaller absolute MDA_GT_ was considered the better, while the other was considered the worse algorithm for that segment. The alternative hypothesis (H1) was that following supervision and adjustments by the physician, segments from the worse algorithm exhibit a larger absolute MDA_GT_ in ≥65% of cases compared to those adjusted from the better algorithm. A flow chart of trial activities is shown in Fig. 1.

### Dosimetric effects

A test segment volume was defined as the volume between the ground truth shell and the test segment from CTV2_algo1_ outside the CTV2_algo1_. Both, the test segment from algo1 and the ground truth segment available as RT-structure DICOM objects, were referenced to the same planning CT and converted into binary volumetric masks in MATLAB (version R2020b, MathWorks Inc., Natick, MA, USA). To extract the volume located between both shells, even in the absence of direct overlap, a morphological envelope approach was applied in MATLAB. A three-dimensional morphological closing operation was applied using the “imclose” function with an ellipsoidal structuring element. The resulting volume was pruned by connected-component labelling (bwconncomp), so that only components in direct spatial contact with both shell surfaces (tested via dilation-based adjacency) were retained. The resulting inter-shell volume was converted back into slice-wise closed planar contours and written to a new RT-structure DICOM object. A margin of 3 mm, 5 mm, and 7 mm was subsequently expanded around the CTV2_algo1_ and the generalized equivalent uniform dose (EUD) was calculated for this test segment volume using a tumour specific parameter a = −20 [20]. The EUD for the volume between the ground truth shell and the STAPLE segment outside STAPLE CTV2, the STAPLE test volume from the adjusted segments by the physicians outside the STAPE consensus CTV2_algo1_, was determined accordingly.

### Statistics

Statistical analysis was performed with SAS statistical software system SAS/STAT 15.1 (SAS Institute Inc, Cary NC, USA). Power and sample size analysis were performed using the Procedure Power from the Statistic-Software SAS® (SAS 9.4, SAS/STAT 14.3, Cary NC) for an exact superiority test for a binomial proportion, with a null proportion of 0.5 and an alternative proportion of 0.65. With a sample size of 130 pairwise comparisons of segments of CTV2_algo1_ and CTV2_algo2_, using at least one segment from the adjusted CTV2 from the 10 selected treatment sessions, supervised by 13 physicians, the power was 0.928 at an alpha level of 0.025.

The exact binomial test for the binomial proportion was performed using the procedure Freq. The pairwise comparison of MDA_GT_ after adjustment was classified as positive, if the absolute MDA_GT_ value of the test segment adjusted from the worse algorithm was greater than that from the better algorithm per supervising physician. If the proportion of positive comparisons was found to be significantly greater than 0.5, than subgroup analysis of the proportion of positive comparisons was sequentially performed for test segments with MDA_GT_ differences between the segments from CTV2_algo1_ or CTV2_algo2_ that were larger vs. equal/smaller than the median MDA_GT_ difference, using Fisher’s exact test.

In a more quantitative analysis, the dMDA_GT_ after adjustment by the physicians was analyzed in dependence on the dMDA_GT_ of the segment by the algorithm before adjustment. A mixed linear model was employed for the analysis, considering that repeated measures were obtained from the same physician (proc mixed, SAS). The respective segment and the physicians were handled as random effect variables. As main classification effects, the respective algorithm was included.

In separate analyses, physicians were grouped into predefined experience groups based on their contouring expertise: In addition, complementary segments to the test segments were analyzed, both of which form the overall surface of the CTV deformed by the considered algorithm. The heterogeneity in the dispersion of the MDA_GT_ values around their median was analyzed per test segment and the generating algorithm using the Ansari-Bradley test adjusted for differences in location (procedure napar1way, SAS) and significant heterogeneity leads to the use of a mixed model. A 2-sided *p*-value of <0.01 was considered as of significant importance.

## Results

### Primary endpoints: MDA_GT_

For the primary endpoint, MDA_GT_ of test segments on CTV2 deformed by algo_1_ and algo_2_ and adjusted by physicians were pairwise compared per physician. Of the 169 pairwise comparisons made, 151 showed that the segment from the algorithm, with a larger MDA to ground truth (MDA_GT_) before adjustment also had the larger MDA_GT_ after adjustment (Table 1). Across all comparisons, the worse-performing algorithm before adjustment remained worse after adjustment in 89.4% of cases. This binominal proportion was significantly greater than 0.5 (exact binomial test, p < 0.0001), with an exact 95% confidence interval of 0.837 to 0.936. Therefore, H0 was rejected, indicating that the MDA_GT_ after adjustment is not independent of the precision of the deformed target structure by the algorithm. Instead, the alternative hypothesis is accepted, that the bias introduced by the algorithm was not fully compensated by the physician. Algo_1_ was the worse algorithm in 11 of the 13 test segments. This result at the primary endpoint was independent from the physician (p = 0.9729, Fisher’s exact test) and the respective patient (p = 0.0803, Fisher’s exact test). There was no significant heterogeneity in the proportion of positive comparisons between segments with an MDA above versus below the median value of 7.07 mm (p = 0.6201, Fisher’s exact test). The binomial proportions of positive comparisons were 0.910 (95% CI: 0.824 – 0.963) and 0.879 (95% CI: 0.812 – 0.946), respectively.

**Table 1:** Analysis of the primary endpoint: The proportion of test segments for which the worse-performing algorithm (Algo_worse_) still shows a larger absolute mean distance to agreement with the ground truth after physician adjustment (adjustMDA_GT_) compared with the adjusted segment from the better-performing algorithm (i.e., positive pairwise comparisons).

		Binomial proportion of positive pairwise comparisons of adjusted CTV-segments
All test segments	adjustMDA_GT_(Algo_worse_) > adjustMDA_GT_(Algo_better_)	151 / 169
Test segments with unadjusted MDA > Median	adjustMDA_GT_(Algo_worse_) > adjustMDA_GT_(Algo_better_)	71 / 78
Test segments with unadjusted MDA ≤ Median	adjustMDA_GT_(Algo_worse_) > adjustMDA_GT_(Algo_better_)	80 / 91

Legend: Test segments with unadjusted MDA> or ≤Median: This pairwise comparison was also performed in dependence on the MDA between unadjusted segments from both deformable registration algorithms, either > or ≤than the median MDA value of 7.07 mm.

Furthermore, we analyzed, whether the adjustments made by the physicians were larger for segments from the worse algorithm compared to segments from the better algorithm in pairwise comparisons. Overall, larger adjustments were performed in 119 out of 169 paired comparisons from the worse algorithm (p < 0.0001, exact binomial test, p = 0.5). There was no significant heterogeneity between physicians in making larger adjustments for the segments from the worse algorithm (p = 0.2651, Fisher’s exact test).

### Quantitative analysis of adjustments

**Fig. 2:**
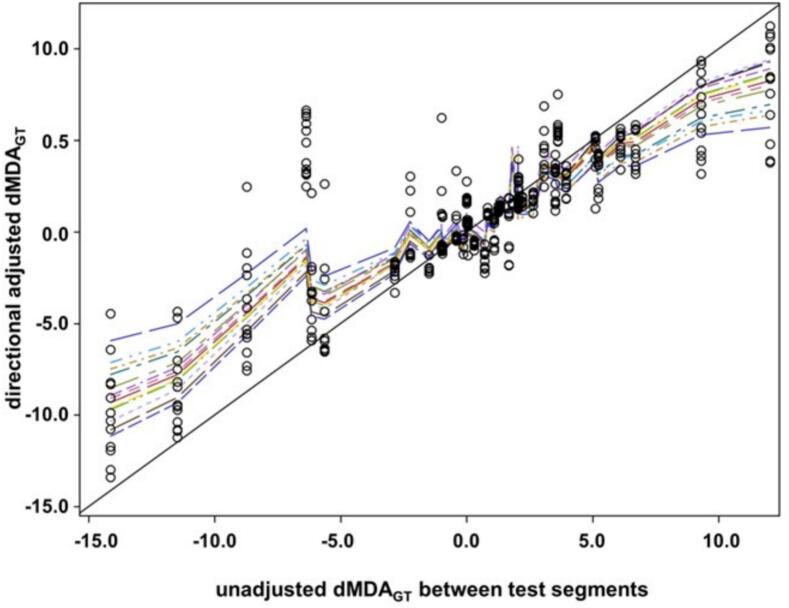
Dependence of the directional MDA of a segment on the surface of a clinical target volume to ground truth (dMDA_GT_) after adjustment by a physician on the dMDA_GT_ of this segment before adjustment. The segments were generated by the three algorithms. Legend: Open circles: observed values; dashed lines: predicted values by a mixed linear model at the observed values connected by drawn or dashed straight lines in different colors in dependence on the supervising physician. For comparison, the drawn line represents 1:1 relation.

In a next step, we analyzed the quantitative dependence of the directional MDA to ground truth values of the test segments, which were adjusted by a physician, in dependence on the dMDA_GT_ values of the unadjusted segments generated by the corresponding algorithm. Fig. 1 summarizes the data from the 13 test segments, generated by each of the three algorithms and the adjustments by the 13 physicians. The dispersion of the dMDA_GT_ values after adjustment by the physicians differed between the test segment algorithm combinations (p < 0.0001, Ansari-Bradley test for scale differences). Therefore, a mixed linear model was used. The dMDA_GT_ values after physicians’ adjustment were significantly dependent on the dMDA_GT_ values for the segments deformed by the algorithms without adjustment (p < 0.0001, F-test). The maximum likelihood estimates of the regression effect of the adjusted on the unadjusted dMDA_GT_ amounted 0.7009 +/- 0.0206 (p < 0.0001, F-Test). An intercept did not become significant. In addition, there was a significant dependence of the slope on the respective physician (p < 0.0001, F-test). The slopes for the different physicians ranged from 0.4317 ± 0.0726 up to 1.0332 ± 0.0726. Therefore, about 57% of the error introduced by the algorithm is corrected by the physicians at best. In Fig. 2, the predicted dMDA_GT_ values by the mixed model were connected by straight lines for the different physicians. There was a significant regression effect of the dMDA_GT_ after adjustment by the physician on the dMDA_GT_ by the algorithm before adjustment (p < 0.0001, F-test). This slope effect was dependent on the supervising physician (p = 0.0003, F-test). Grouping physicians according to predefined experience categories, such as contouring expert with over 4 years (group A) of experience in contouring, physicians with 2–4 years of experience (group B) in contouring, and those with 0.5–2 years of experience (group C), revealed a moderate experience-effect on slope. In particular, there was a decrease in slope by −0.1218 +/-0.0407 or −0.1345 +/-0.0407 (p = 0.0005, F-test) for Group A and B physicians, respectively, compared to Group C physicians.

The residuals of the observed dMDA_GT_ values after adjustment from the mixed model had a standard deviation of 2.02 mm. A carryover effect depending on the algorithm by which the considered segment in a preceding reviewing session was deformed did not become significant using the mixed model, neither as a main effect nor as a slope-modifying effect (p > 0.01, F-test). Similarly, a washout time effect from the last to the considered reviewing session did not became significant as a regression effect (p > 0.01, F-test).

In addition, we analyzed the performance of the STAPLE consensus volumes for each segment from algorithms (I) and (II), generated from the respective adjusted segments of all physicians. Using the mixed model, the STAPLE consensus volume exhibited a comparable performance to the maximum likelihood estimate of the dMDA_GT_ values from all physicians. Furthermore, the dependence of the dMDA_GT_ values of the staple consensus volumes on the dMDA_GT_ values of the unadjusted test segments showed a similar but numerically better slope of 0.6184 +/- 0.0951 compared to the maximum likelihood estimate for the dependence of the dMDA_GT_ values of the adjusted segments by the group of all physicians (p = 0.6951, F-test).

### Complementary CTV segments

**Fig. 3:**
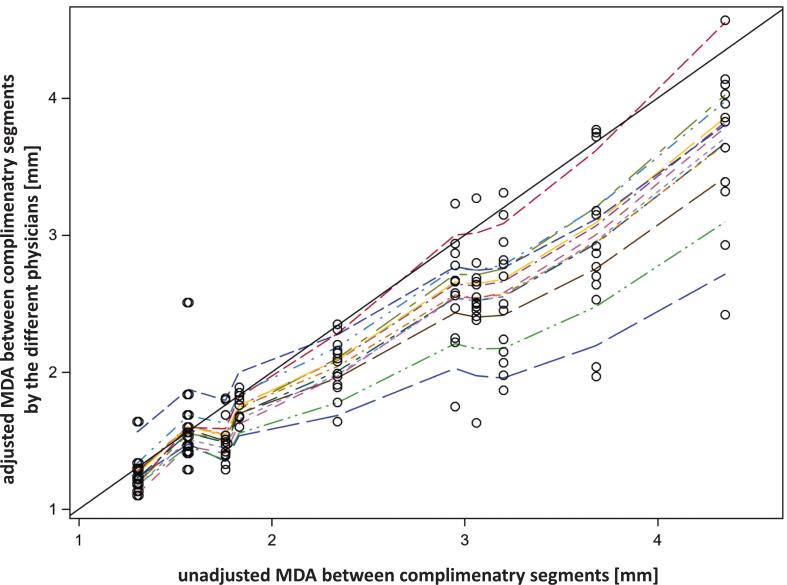
Absolute MDA values between corresponding complementary segments on the CTV2 deformed by the contour-based and the hybrid algorithms after adjustments by the physicians in dependence on the MDA values between the unadjusted segments. Legend: The dashed lines connect predicted absolute MDA values by the mixed model for the adjusted segments by the different physicians. The drawn 1:1 line is given as a reference. The drawn circles represent measured MDA values between adjusted complimentary segments from both algorithms by the different physicians.

For each test segment, the complementary segment was analyzed making up the overall surface of the CTV2 deformed by the considered algorithm. The median absolute MDA value between the deformed CTV surfaces by algo_1_ and algo_2_ on CBCT2 was 1.83 mm (range 1.30–4.35 mm). Given the absence of the ground truth for the complementary segments, MDA values between complementary unadjusted segments generated by algo_1_ and algo_2_ were computed. These values were then compared with the MDA values for the respective adjusted complementary segments by the physicians. A prerequisite for an adjustment toward ground truth by the physicians is, that the MDA values between test segments from both algorithms tend to 0 mm, i.e the same volume, after adjustment. Analysis by a mixed model revealed, that the MDA values between the adjusted complementary segments exhibited a positive association with the MDA values between unadjusted complimentary segments (p < 0.0001; F-test). The maximum likelihood estimate for the slope of the relation of the MDA values after adjustment on the MDA before adjustment was 0.7526+/-0.0376 (95% CI; 0.6784 – 0.8269). Fig. 3 shows, that there was a significant dependence of this slope on the physician (p < 0.0001, F-Test). The intercept did not become significant. Therefore, physicians’ adjustments reduced the MDA between complementary segments from both algorithms by a similar coefficient observed for the reduction in dMDA_GT_ values of the test segments.

### Dosimetric effects of the CTV deformations

The EUD values in the ground truth test segment volumes outside the CTV2 deformed by the worse algorithm with the largest MDA_GT_, algo1, were below 95% in 38% of the test segment volumes using a PTV margin of 3 mm and the clinical dose distribution. In comparison to the EUD values for the test segment volumes, the EUD-values for the respective STAPLE test volumes after adjustment by the physicians increased significantly (p = 0.016, signed rank test), leaving 23%, 15% and 8% of the STAPLE test volumes at an EUD value <95% at PTV margins of 3 mm, 5 mm, and 7 mm. As the STAPLE test volumes were rather small with a median value of 1.4% of the respective STAPLE CTV2, the dosimetric effects of the cold spots in the STAPLE test volumes are further diminished by the majority of the STAPLE CTV at an EUD >95%.

## Discussion

Online onboard ART is a promising new technique that ensures the prescribed dose covers the interfractionally deformed CTV, while simultaneously reducing the PTV margins around the clinical target volume. Thus, it is possible to spare dose to the nearby organs at risk in a whole range of anatomic areas from head and neck to the pelvic region [12], [20], [21], [22], [23], [24]. A recent randomised phase II trial of dose-escalated adaptive radiotherapy in patients with bladder cancer using a hybrid adaptive workflow has effectively demonstrated the potential of dose escalation as an organ-preserving multimodality treatment option [25]. The initial results show a low salvage cystectomy rate and overall survival similar to that seen in cystectomy cohorts at a low risk of late grade ≥3 side effects [25].

Meanwhile, our present study results reveal that the quality of contours based on AI-generated influencer structures is of crucial importance for a proper online onboard navigation in adaptive radiation therapy (ART), as errors may bias clinical workflow and contouring. The quality of the automatically generated contours was quantified by the mean distance (MDA) to the true segment. The median MDA between the examined AI-algorithms was 1.83 mm. Similar MDA values of 1–4 mm were found in other studies for the accuracy of algorithms for bladder, rectum or prostate contour propagation between different imaging modalities compared with manual contouring [26], [27].

A major finding from the present study is that the residual mean distance to ground truth of the selected test segments after manual adjustment by the physician is not negligible. On the contrary, physicians reduced the residual MDA_GT_ on average to 70% and at best to 43% of the deviation from ground truth caused by the respective deformation algorithm. Consequently, the PTV for an algorithm-generated CTV in online adaptive radiotherapy should include an additional component accounting for residual propagation errors that are not corrected by the observer. Whether this residual error is systematic or random depends on the similarity of the anatomic scenarios captured by the source and the target CT image data set, the shape and location of the CTV itself, and the respective deformation algorithm. For the treatment series, underlying the present study, a systematically smaller bladder volume was observed on CBCT1 than on the planning CT with increasing bladder volume increases from CBCT1 to CBCT2 [1], [20].

For image-guided radiotherapy, anatomic changes from planning CT to CBCT1 are accounted for by an internal margin [28]. In bladder cancer, PTV margins of 7–15 mm in the superior direction are often used. These margins consist of internal margins and setup margins [2], [25]. Image guided radiotherapy is equivalent to using a copy algorithm for CTV propagation from planning CT to CBCT1 without adjustments of the CTV by a human adapter. Based on the results of the present study, this internal margin could be reduced to 70% of its size for the IGRT value by online adaptation for bladder cancer on average, if the results for the test segments would be generalizable to the whole CTV. The dosimetric consequences of the tendency of the physicians to under-correct the test segments from the worse DIR algorithm for bladder cancer CTV propagation was quantified. To achieve an EUD value >95% in more than 90% of the STAPLE consensus volumes, a PTV margin of 7 mm was necessary for the bladder CTV.

A rigid propagation algorithm or a CT-slice wise rigid propagation algorithm is frequently used in the CBCT-guided online adaptive radiotherapy workflow [29], [30]. Target volume propagation algorithms based on artificial intelligence are evolving, as discussed above. With these developments, propagation errors of the CTV will decrease in the future.

The accuracy of algorithms is usually evaluated by comparison with a manually delineated contour [31], anatomic landmarks [32], [33] or to a digital [34] or physical phantom [35]. Many studies use manually contoured anatomical contours by one observer as ground truth. However, the accuracy of the manual contours was usually not put into question [31]. In other studies, considering inter-physician delineation variability, a consensus structure was selected as reference [31], [36], [37]. However, consensus volumes between human observers do not necessarily represent the ground truth, especially if the observers are biased by predefined contours with errors as shown in the present study. That human’s decisions are subtle to incorrect information is also known from other fields of exposure to artificial data. A recent randomized controlled trial which showed that exposure to lower-quality information retrieved by online research engines is associated with higher probability of believing misinformation of highly popular news articles directly after publication [38]. Deepfakes represent images, records or video material, which are manipulated with the help of deep learning techniques [39]. More and more, deepfake may pose a challenge for society, not only in media, but also in other transeunt and social areas. Studies examining human capacity to identify deepfakes showed that it is increasingly difficult to discriminate deepfake from reality [39], [40], [41], [42]. AI-synthesized images are possibly perceived as authentic, sometimes even more than real images [40].

Other studies compared auto-contoured organs at risk, but not target volumes, with the respective manual organs at risk in various regions of the body and with different ground truth standards [42], [43], [44], [45], [46], [47], [48], [49], [50], [51], [52]. There exist some observations that inter-observer variability of pelvic contours tend to be smaller, if observers adjust structures generated by AI than contouring without computational assistance [52], [53]. This points to a tendency of the observers to follow the contours obtained from the AI-algorithm. The dependency of this tendency on the accuracy of the automated contours was not analysed in preceeding studies.

Contrary to the aforementioned the present study uses another design. We considered the clinical target volume instead of organs at risk. The CTV comprises not only the gross tumor volume, but also the surrounding tissue that has a higher risk of microscopic spread and therefore does not follow strictly anatomic borders. All parts of the target volume should be treated with a sufficient dose, as cold spots can dominate the effectiveness of a dose distribution [54]. From a prospective data base of dose fractions, we looked selectively at dose fractions from bladder cancer patients for which propagated target volumes by two algorithms differed by more than 5 mm in segments of their contour. The CTV for focal bladder cancer radiotherapy does not always strictly follow the bladder contour due to extravesical tumor spread and contains only parts of the bladder wall around the tumor within a given margin, defined on the planning CT. Therefore, it is more complex than the bladder contour itself. We used errors in automatically generated and propagated CTV structures from ground truth as an input and tested the performance of the physicians to mitigate the errors introduced by the auto-contouring algorithms during supervision of the target volumes. The hypothesis, that the accuracy of the edited CTV by the clinician is independent from the accuracy of the CTV auto-contour could be rejected in this study.

In the following, a range of limitations shall be discussed. It is a mono-institutional study not fully covering the heterogeneity in the training of physicians across multiple institutions. The number of CTV segments analysed is limited, but we gave importance to the fact that the different segments were identified by deviations from two deformable registration algorithms that could also be clinically used and were not manually deformed. While the general tendency, that MDA_GT_ of segments deformed by the worse algorithm remains worse after adjustment by a physician, could be supported by this study, there may be special sites in the pelvic area, where quantitative results may differ. This issue has to be analysed in further studies.

Finally, the question remains of the dosimetric impact and clinical implications of the present results on a fractionated radiotherapy series. If errors introduced by contour-generation algorithms are randomly distributed across the target surface from fraction to fraction, their effects will be diminished during fractionation [20].

## Conclusions

The present study shows, that the accuracy of supervised structures strongly depends on the accuracy of the AI generated contours before supervision. Errors inherent in complex target volumes by contour generating algorithms may bias final treatment schedules. Computational automation bias of physicians compromises the precision of ART for complex CTV-structures.

## Ethics declarations

All treated patients gave their consent to the treatment taking part in the prospective, institutional clinical registry trial (18-8364-BO). The study was conducted in accordance with the principles of the Declaration of Helsinki. The study was approved by the Ethics committee of University Hospital Essen of University of Duisburg-Essen (23-11595-BO).

## CRediT authorship contribution statement

**Nika Guberina:** Writing – review & editing, Conceptualization. **Aymane Khouya:** Conceptualization, Validation, Funding acquisition. **Christian Hoffmann:** Funding acquisition. **Gerrit Fischedick:** Conceptualization. **Yasemin Alberti:** Funding acquisition. **Julian Hlouschek:** Funding acquisition. **Fabian Freisleben:** Funding acquisition. **Andreas Herz:** Conceptualization. **Mike That Troung Ton:** Funding acquisition. **Lars Oliver Kiwitt:** Funding acquisition. **Ezgi Ceren Sahin:** Funding acquisition. **Alina Santiago Garcia:** Conceptualization. **Thomas Gauler:** Funding acquisition. **Christoph Pöttgen:** Conceptualization. **Maja Guberina:** Writing – review & editing, Conceptualization. **Martin Stuschke:** Writing – review & editing, Formal analysis, Investigation.

## Funding

This research did not receive any specific grant from funding agencies in the public, commercial, or not-for-profit sectors.

## Declaration of competing interest

The authors declare that they have no known competing financial interests or personal relationships that could have appeared to influence the work reported in this paper.

## Data Availability

The datasets used and/or analysed during the current study are available from the corresponding author on reasonable request.
